# Rimonabant and Cannabidiol Rewrite the Interactions between Breast Cancer Cells and Tumor Microenvironment

**DOI:** 10.3390/ijms241713427

**Published:** 2023-08-30

**Authors:** Maria Chiara Proto, Donatella Fiore, Maurizio Bifulco, Patrizia Gazzerro

**Affiliations:** 1 Department of Pharmacy, University of Salerno, 84084 Fisciano, SA, Italy; maproto@unisa.it (M.C.P.); dfiore@unisa.it (D.F.); 2 Department of Molecular Medicine and Medical Biotechnologies, University of Naples “Federico II”, 80131 Naples, NA, Italy; maubiful@unina.it

**Keywords:** rimonabant, cannabidiol, breast cancer, tumor microenvironment, metastatic breast cancer

## Abstract

The spread of breast cancer to distant sites is the major cause of death in breast cancer patients. Increasing evidence supports the role of the tumor microenvironment (TME) in breast cancers, and its pathologic assessment has become a diagnostic and therapeutic tool. In the TME, a bidirectional interplay between tumor and stromal cells occurs, both at the primary and metastatic site. Hundreds of molecules, including cytokines, chemokines, and growth factors, contribute to this fine interaction to promote tumor spreading. Here, we investigated the effects of Rimonabant and Cannabidiol, known for their antitumor activity, on reprogramming the breast TME. Both compounds directly affect the activity of several pathways involved in breast cancer progression. To mimic tumor–stroma interactions during breast-to-lung metastasis, we investigated the effect of the compounds on growth factor secretion from metastatic breast cancer cells and normal and activated lung fibroblasts. In this setting, we demonstrated the anti-metastatic potential of the two compounds, and the membrane array analyses highlighted their ability to alter the release of factors involved in the autocrine and paracrine regulation of tumor proliferation, angiogenesis, and immune reprogramming. The results enforce the antitumor potential of Rimonabant and Cannabidiol, providing a novel potential tool for breast cancer TME management.

## 1. Introduction

In recent years, the study of tumors has focused not only on the alterations of the neoplastic cells but also on the modifications of the whole cellular milieu. The biological implications of the tumor microenvironment (TME) on all stages of tumorigenesis, progression, and metastasis have been reported [1]. The TME includes the extracellular matrix (ECM) and several cell types such as fibroblasts and immune and endothelial cells. In this context, fibroblasts have been suggested to play a pivotal role in tumor development. Fibroblasts are quiescent under normal conditions, but due to their ongoing crosstalk with tumor cells, they can become activated and acquire a tumor-supporting phenotype. Indeed, the release of factors such as PDGF, TGF-β, FGF, and MMPs by tumor or stromal cells induces fibroblasts to acquire contractile capacities and to express specific markers such as α-SMA, FAP, and FSP, characteristic of the activated phenotype [2]. Once activated, fibroblasts can form myofibroblasts or cancer-associated fibroblasts (CAFs), which proliferate and in turn secrete soluble factors able to support tumor progression by remodeling tissue architecture, repressing local immune responses, and increasing migration and invasion [3]. CAFs, a highly heterogeneous stromal cell population, are predominant components of the microenvironment in most solid tumors, including breast cancer. The presence of CAFs in the microenvironment correlates with a poor prognosis, with resistance to therapies and disease recurrence in several tumor patients [4]. Different CAF subpopulations have been identified in several cancers. In particular, four subsets of CAFs (S1–4) have been reported in breast cancer subtypes (Luminal A, HER2, and triple negative), each of which corresponds to a prognostic impact [5]. More recently, four main subsets of CAFs, two of which are associated with T-cell exclusion, have been identified in lung cancer [6]. During tumor growth, the interplay between tumor and stromal cells in the TME is orchestrated by various regulatory molecules, including transcription and growth factors able to affect the expression of several genes involved in the metastatic process [7,8]. In the primary tumor and pre-metastatic niche, hypoxia-inducible factors (HIFs), by regulating the expression of many genes in cellular components of the TME, induce intra- and extravasation through the production of angiogenic factors. It is noteworthy that the expression of HIF-1 target genes was increased in triple-negative breast cancer, and a reduction in breast cancer lung metastases was related to HIF-1 inhibition [9]. In the tissue surrounding the niche, tumor cells are able to induce inflammation through several transcription factors, including NfKB and TGF-β. Specifically, NfKB activation has been associated with breast cancer progression, and high levels of TGF-β were related to a poor prognosis and drug resistance in breast cancer patients [10]. In this context, drugs able to directly target CAF subtypes, inducing their reprogramming, and/or to indirectly interfere with TME modifications represent an appealing strategy to improve anticancer therapies.

A growing amount of evidence reports the involvement of the endocannabinoid system in cancer modulation, affecting proliferation, migration, invasion, and metastases [11,12,13,14,15]. Furthermore, modulation of the endocannabinoid system has been reported to interfere with components of the TME, although its role in tumor–stroma interactions is unclear [16].

The anticancer properties of Rimonabant (SR141716A, here referred to as SR) and Cannabidiol (CBD), two well-known CB1 receptor antagonists/inverse agonists, have been reported in a large number of cancer types, but their actions are not always related to the direct modulation of the endocannabinoid system [11,16]. In particular, we reported that SR exerts its antitumor effects through a CB1-independent epigenetic mechanism, directly interacting with the p300-histone acetyltransferase domain [12]. Moreover, SR controls Wnt/β-Catenin pathway activation both in in vitro and in vivo models of colorectal cancer and in colon cancer stem cells (CSCs), without affecting healthy colon epithelial cells, suggesting its potential selectivity toward cancer cells [12,13]. Of note, CBD has been reported to modulate the TME by affecting cytokine production in breast cancer cells and thereby reducing macrophage recruitment to tumor sites [17].

Starting from the previous studies, in this work we evaluate the potential of SR and CBD in the control of TME and related processes, focusing on their ability to modify the secretome during the breast cancer–stroma crosstalk.

## 2. Results

### 2.1. SR and CBD Control Transcriptional Activation of Tumor-Related Pathways

**Figure 1:**
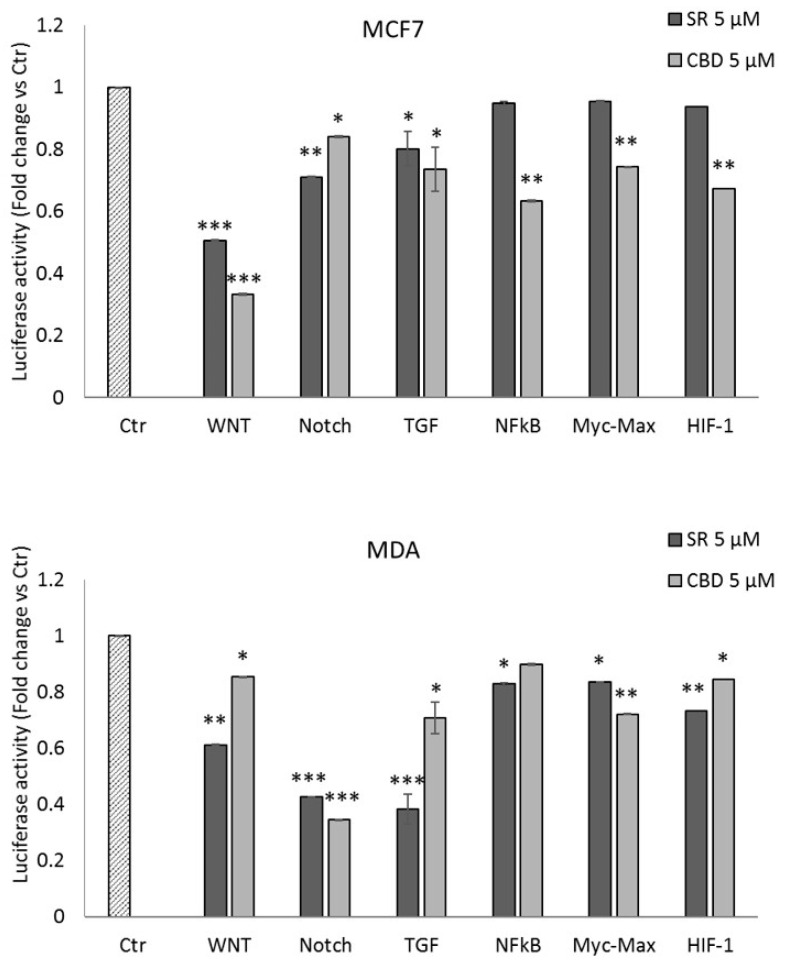
Luciferase activity on selected cancer-related Transcriptional Responsive Elements (TREs). Analysis of luciferase activity of TREs controlled by Wnt (TCF/LEF), Notch (RBP-Jκ), TGFβ (SMAD2/3/4), NfkB, Myc/Max, and HIF-1α in the MCF7 and MDA-MB-231 cell lines. The histograms represent luciferase activity measured at 24 h from transfection with a reporter construct containing the TRE elements and treated with vehicle, SR, or CBD 5 μM for 18 h. Firefly luciferase was normalized to Renilla luciferase reading, and the data were plotted as fold change (mean ± SD of four independent experiments in triplicate; unpaired two-tailed Student’s *t*-test; * *p* < 0.05, ** *p* < 0.01; *** *p* < 0.005) compared to control cells.

Both SR and CBD were found to be able to inhibit tumor cell proliferation with several mechanisms [11,12,13,14]. In this work, we focused our attention on the ability of SR and CBD to control the tumor microenvironment dynamic in breast cancer cells. Through cell viability (MTT) and cell proliferation (BrdU incorporation) assays, we identified for both compounds a lower (0.5 µM) and a higher (5 µM) concentration, significantly cytostatic but not cytotoxic (Figure S1). Then, 5 µM of SR and CBD was used to evaluate luciferase activity on the Transcriptional Regulatory Elements (TREs) of several transcription factors involved in the control of tumor-related pathways in MCF7 and MDA-MB-231 cell lines. Both compounds significantly inhibit the transcriptional activity of TCF/LEF (Wnt pathway), RBP-Jk (Notch pathway), SMAD2/3/4 (TGFβ pathway), NfKB, Myc-Max, and HIF1α factors in MCF7 and MDA-MB-231 cell lines (Figure 1).

**Figure 2:**
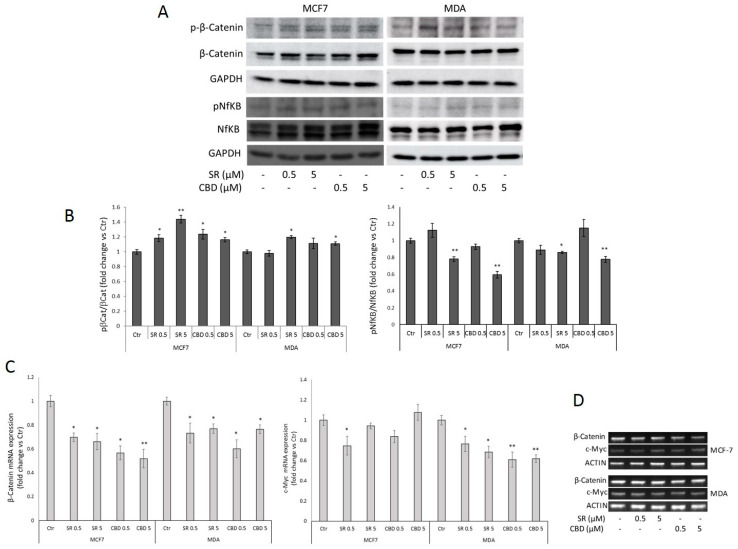
Representative Western Blots (**A**) and densitometric analyses (**B**) of β-Catenin (total and Ser33/37-phosphorylated forms) and NfKB (p65, total and Ser536-phosphorylated forms) in MCF7 and MDA-MB-231 cells treated with vehicle, SR, or CBD (0.5 or 5 μM) for 24 h. GAPDH was used as a loading control. (**C**,**D**) β-Catenin and c-Myc RT-PCR analysis in MCF7 and MDA-MB-231 cells treated with vehicle, SR, or CBD for 24 h. Representative images of β-Catenin and c-Myc mRNA expression are shown in (**D**). ACTIN was used as a reference gene. Data are expressed as mean ± SD of at least three independent experiments. * *p* < 0.05, ** *p* < 0.01 vs. control.

In agreement with our previous studies in CRC, SR and CBD induce an increase in β-Catenin-Ser33 phosphorylation (tag of degradation) and a reduction in β-Catenin mRNA levels, confirming the inhibition of the Wnt/β-Catenin pathway (Figure 2A,B). Surprisingly, despite the slight promoter inhibition, we found that in both the MCF7 and MDA-MB-231 cells, SR and CBD significantly inhibit Ser536 phosphorylation of NfKB, corroborating their anti-inflammatory activity in tumor cells (Figure 2A,B). The analysis of mRNA expression substantially confirms c-Myc pathway inhibition only in MDA-MB-231 cells, starting from low concentrations of both SR and CBD (Figure 2C,D).

**Figure 3:**
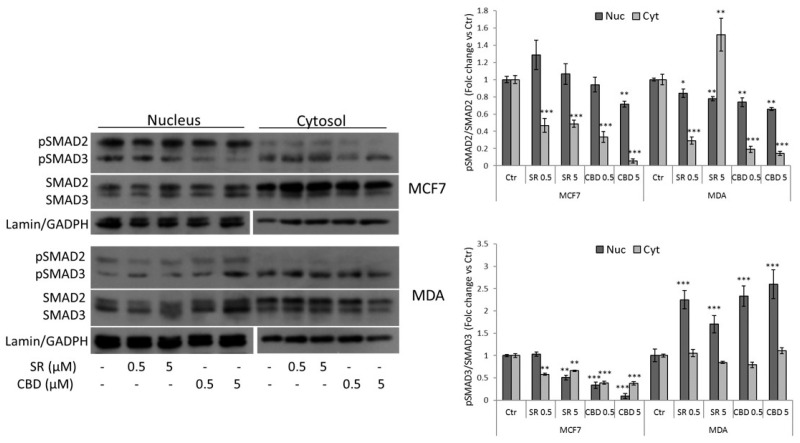
Representative Western Blots and densitometric analyses of SMAD2/3 (total and phosphorylated forms) in nuclear and cytosolic protein fractions obtained from MCF7 and MDA-MB-231 cells treated with vehicle, SR, or CBD (0.5 or 5 μM) for 24 h. Lamin and GAPDH were used as a loading control of nuclear and cytosolic fractions, respectively. Data are expressed as mean ± SD of at least three independent experiments. * *p* < 0.05, ** *p* < 0.01, *** *p* < 0.005 vs. control.

Canonical TGFβ signaling transduction from membrane receptor to the nucleus involves SMAD family proteins. Once active, TGFβR recruits and phosphorylates SMAD2 and SMAD3 in the cytoplasm, which in turn complex with SMAD4 and translocate to the nucleus and bind DNA to responsive elements [18]. To confirm the data from the luciferase assay, we fractionated nuclear and cytosolic proteins to analyze the dynamic of SMAD transcriptional factors after SR and CBD treatments. The results highlighted that in MDA-MB-231 cells, both SR and CBD reduce the amount of nuclear phospho-SMAD2 but not phospho-SMAD3 that otherwise accumulates in the nucleus (Figure 3). On the contrary, in MCF7 cells, the compounds reduce the nuclear and cytosolic amount of phospho-SMAD3. In this cell line, the expression of phospho-SMAD2 was reduced only in the cytosolic fraction and in the nucleus only after treatment with CBD 5 µM (Figure 3). Overall, despite the different trend between the two cell lines, this result enforces the observation that our compounds impair TGFβ signaling.

### 2.2. Conditioned Media from SR- or CBD-Treated Cells Controls Tumor Cells Proliferation and Lung Fibroblast Activation

**Figure 4:**
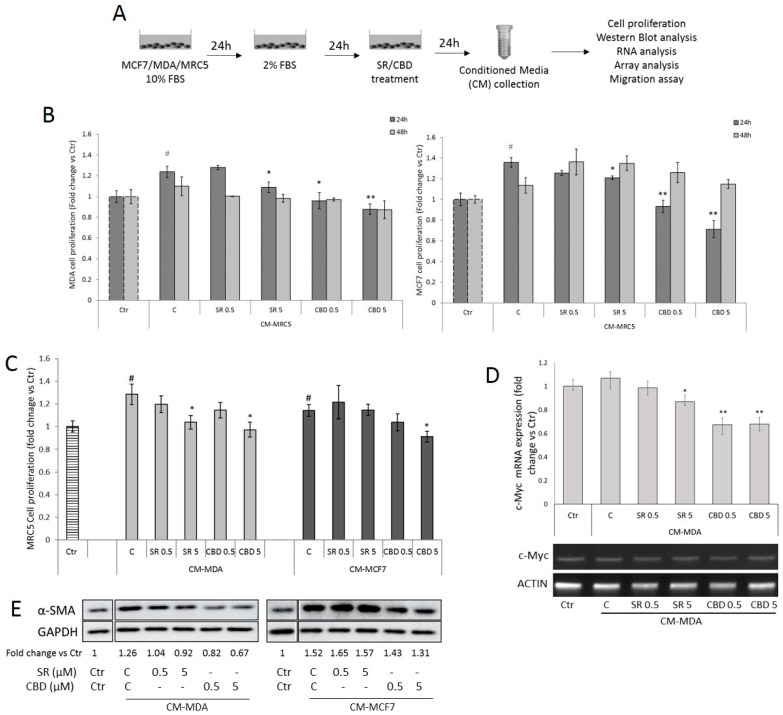
(**A**) Schematic representation of experimental settings for collection of conditioned media (CM) from breast cancer cell lines or human lung fibroblasts. (**B**) Cell proliferation assay was used to evaluate the effect of CM from MRC5 (CM-MRC5) untreated or treated with SR or CBD for 24 h at the indicated concentrations in MDA-MB-231 and MCF7 cells or (**C**) proliferation assays performed in MRC5 cells cultured with CM from breast cancer cell lines (CM-MDA and CM-MCF7) untreated or treated with SR or CBD for 24 h at the indicated concentrations. (**D**) Representative c-Myc mRNA expression (lower panel) and densitometric analysis (upper panel) in MRC5 cells untreated (Ctr) or exposed to CM from MDA-MB-231 (CM-MDA) obtained as in panel C. ACTIN was used as a reference gene. (**E**) Representative Western Blots and densitometric analyses (fold change vs. control) of α-SMA protein expression in MRC5 cells untreated (Ctr) or exposed to CM-MDA or CM-MCF7 obtained as in panel C. GAPDH was used as loading control. Data are expressed as mean ± SD of at least three independent experiments (# *p* < 0.05 vs. untreated basal Ctr; * *p* < 0.05, ** *p* < 0.01 vs. untreated control (C) from CM).

The results described above suggest that SR and CBD strongly influence key pathways involved in tumor-related mechanisms, including cancer stemness, chemoresistance, metastasis, immune response, and generally tumors [19]. Metastatic dissemination results from a fine crosstalk between the tumor microenvironment, cancer cells, and the stroma that directly regulates and prepares the niche for tumor cell colonization. The lungs are among the principal secondary sites for breast cancer metastatic cells [20,21]. In order to study the possible role of SR and CBD in tumor–stroma communications, we collected conditioned media (CM) from MCF7 cells, metastatic MDA-MB-231 cells, and normal human fibroblast MRC5 cells, as schematized in the Figure 4A. First, we established the optimal concentrations of SR and CBD to treat MRC5 normal fibroblast and, as for breast cancer cells, we chose non-toxic concentrations of 0.5 µM and 5 µM for both compounds (Figure S2). We next assessed if the CM from MRC5 (CM-MRC5) cells treated with the vehicle or 0.5 µM and 5 µM of SR or CBD for 24 h affect tumor cell proliferation. Compared to the basal control (Ctr), the CM from untreated MRC5 significantly induce the proliferation of both MCF7 and MDA-MB-231 cells. However, the CM from both SR- and CBD-treated fibroblasts negatively regulate breast cancer cell proliferation, with a greater effect at high concentrations and a strongest efficacy in non-metastatic MCF7 cells (Figure 4B). It is noteworthy that through the secretion of several players, such as chemokines, cytokines, and growth factors, cancer cells are responsible for fibroblast activation and transformation in cancer-associated fibroblasts (CAFs). We then evaluated if the treatment with our compounds affects lung fibroblast proliferation and activation. The CM from both MCF7 (CM-MCF7) and metastatic MDA-MB-231 (CM-MDA) cells induce MRC5 cells proliferation. Interestingly, the contact of fibroblasts with the CM from MDA-MB-231 cells treated with SR or CBD (5 µM) reverts the proliferation induced by untreated CM-MDA after 24 h of treatment. However, only the CM from MCF7 treated with CBD but not with SR significantly reduce MRC5 cell proliferation (Figure 4C). In order to analyze if fibroblast proliferation was associated with the modified expression of fibroblast marker activation, we examined the levels of α-SMA protein. As shown in Figure 4C,E, the trend of α-SMA reflects the changes in cell proliferation. In particular, the treatment of MRC5 with the CM from both cell lines treated with our compounds reverts the induction of α-SMA, suggesting that SR and CBD could prevent the activation and transformation of normal lung fibroblasts. It has been reported that metastasis-associated fibroblasts change their functional roles during the metastatic process and, among others, inflammatory response genes (e.g., TNFα or IL-6) and some transcription factors genes, like c-Myc, are highly upregulated [22]. Here, we found that c-Myc gene expression in MRC5 cells is significantly reduced when exposed to CM from breast metastatic MDA-MB-231 cells treated with SR and, in particular, with CBD (Figure 4D).

### 2.3. SR and CBD Impact Breast Cancer Cell Migration

**Figure 5:**
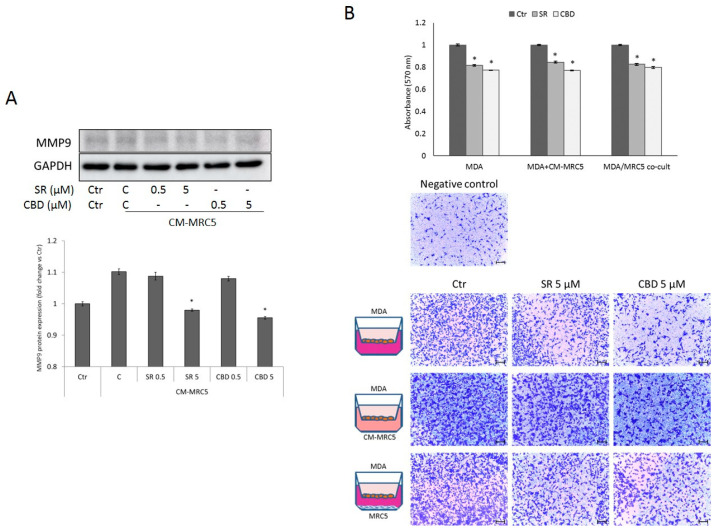
(**A**) Representative Western Blots and densitometric analyses of MMP9 protein expression in MDA-MB-231 cells untreated (Ctr) or exposed to CM-MRC5, obtained from MRC5 untreated or treated with SR or CBD for 24 h at the indicated concentrations. GAPDH was used as loading control. Data are expressed as mean ± SD of at least three independent experiments (* *p* < 0.05 vs. untreated control (C) from CM). (**B**) Transmigration assay of MDA-MB-231 cells treated with SR and CBD (5 μM) for 24 h in different experimental conditions, as depicted in the left panel. The images (right panels) are representative of crystal violet staining (Scale bar 100 µm). The upper histograms represent the absorbance after acetic acid elution of crystal violet. Data are expressed as mean ± SD of at least three independent experiments. * *p* < 0.05 vs. control (C).

The finding that MRC5-derived CM affect breast cancer cell proliferation prompted us to investigate the effect of SR and CBD on breast cancer cell migration. We then analyzed MMP9 expression in the metastatic MDA-MB-231 cells after treatment with MRC5-derived CM. As shown in Figure 5A, the amount of active MMP9 is significantly reduced in MDA-MB-231 exposed to CM-MRC5 treated with SR or CBD (5 µM), compared to CM-MRC5 derived from untreated cells (C), suggesting their potential ability in cell migration control. Moreover, we assessed cell migratory potential using a Transwell system in different conditions. Specifically, we evaluated the anti-migratory potential of SR and CBD in mono-cultured metastatic MDA-MB-231, in cells exposed to CM from treated or untreated MRC5 or when co-cultured with MRC5 (Figure 5B). As expected, when exposed to MRC5-derived CM or co-cultured with MRC5, MDA-MB-231 cell migration increases. However, in all the systems examined, to a similar extent, both SR and CBD significantly reduce MDA-MB-231 cell migration (Figure 5B). The result obtained suggests that, despite being slight, the SR- and CBD-mediated inhibition of breast cancer cell migration is verified even when the cancer cells are exposed to chemotactic factors contained in MRC5-derived conditioned medium or secreted by the co-cultured activated fibroblast.

### 2.4. SR and CBD Modify Growth Factor Contents in Culture Media

It is noteworthy that the tumor microenvironment is an essential player in the metastatic process, supporting and orchestrating the major pathways involved in metastasis formation, tumor cell colonization, vascularization, and all the mechanisms facilitating pre-metastatic niche formation, including the recruitment and activation of immune cells and the alteration in the extracellular matrix (ECM) composition [23,24]. In recent years, the targeting of the tumor microenvironment has become an interesting therapeutic approach. Based on the results previously obtained, we investigated the changes triggered by SR and CBD in the secretome composition of breast cancer cells and lung fibroblasts. To this aim, a membrane protein array of tumor-related growth factors was used to identify the main altered factors secreted by metastatic MDA-MB-231 and MRC5 cells after treatment with 5 µM SR or CBD for 24 h. Several growth factors were significantly modulated by the treatments (Figures S3 and S4), including, among others, some growth factors notably involved in breast cancer, breast-to-lung metastasis, and lung pre-metastatic niche formation. In particular, in MDA-MB-231 and MRC5 cells, SR and CBD affect the secretion of growth factors involved in proliferation and angiogenesis (bFGF, FGF4, -6, -7, VEGF-D, PIGF, PDGF-AB, and -BB), tumor metabolism (IGFII, IGBPs), and immune response (GCSF, MCSF, TGF family members) (Figures S3A,B and S4A,B). Interestingly, in MRC5 cells, both compounds significantly reduce the secretion of TGF-α, -β, -β2, and -β3 (Figure S4A,B). In MDA-MB-231, according to the results from the luciferase assay, SR and CBD inhibit the secretion of TGFα and -β, while only SR reduces the levels of TGFβ2 (Figure S3A,B).

All the factors identified are fundamental players in cancer progression and the metastatic process, so they can profoundly modify tumor–stroma interactions during all the steps of the metastatic cascade. To deeply understand the mechanism of SR and CBD in modulating the microenvironment, we also analyzed the changes in secreted growth factors of MDA-MB-231 cells exposed for 24 h to CM from MRC5 untreated or treated with SR and CBD for 24 h and, vice versa, of MRC5 cells exposed for 24 h to CM from MDA-MB-231 untreated or treated with the two compounds for 24 h. The results highlighted that the contact of MDA-MB-231 cells with CM from MRC5 significantly upregulated the secreted levels of SCF and FGF family members; however, these were reduced when the cells were exposed to CM from MRC5 treated with the compounds. Moreover, also in these conditions, we noticed that IGFBPs were significantly upregulated in MDA-MB-231 exposed to CM from MRC5 treated cells, compared to untreated cells (Figure S3C,D). Interestingly, despite that the conditioned media from untreated MRC5 cells did not increase TGFβ levels in MDA-MB-231 cells, CM from MRC5 treated with CBD significantly decrease its levels (Figure S3D).

It is noteworthy that within the tumor microenvironment, through a multitude of secreted factors, including cytokines, chemokines, and growth factors, cancer cells activate normal fibroblast to induce a CAF phenotype [23]. In our settings, as expected, in agreement with the α-SMA increase, suggestive of a transformation to a CAF-like phenotype, MDA-MB-231 CM induce an upregulation of several MRC5-secreted factors, including EGF and HB-EGF, GMCSF, FGFs, IGFI, IGFBPs, and PDGF-AB. A slight but not significant increase in TGFβ and TGFβ2 was also found (Figure S4C,D). On the contrary, in MRC5 exposed to CM from MDA-MB-231 treated with SR or CBD, many of these factors were significantly reduced compared to untreated CM-MDA, including TGFβ, TGFβ2, IGFI, PDGF-AB, and FGFs (Figure S4C,D), enforcing the observation that the treatment of MDA-MB-231 with SR and CBD prevents the activation of normal fibroblasts in the tumor microenvironment.

**Figure 6:**
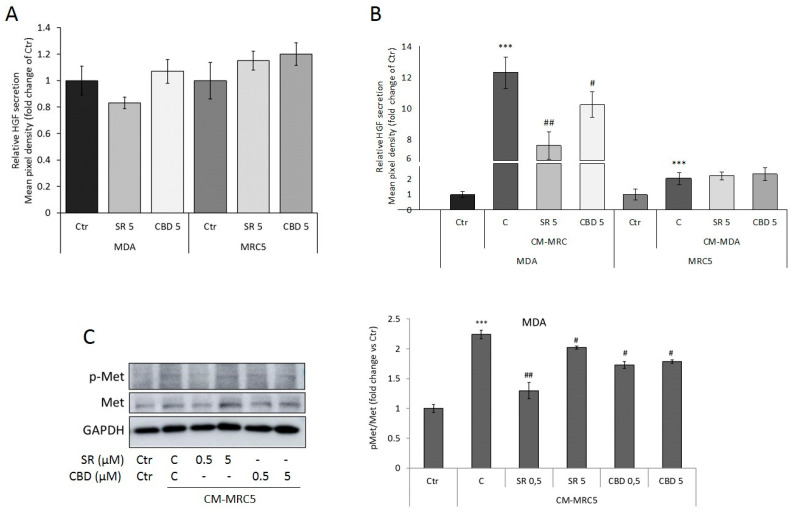
HGF secreted levels in culture media from MDA-MB-231 or MRC5 cells treated for 24 h with SR or CBD 5 μM (**A**) and with CM as indicated (**B**). Histograms represent densitometric analyses of the membrane spots on the antibody array. (**C**) Representative Western Blots and densitometric analyses of c-Met total and phosphorylated forms. GAPDH was used as loading control. Data are expressed as mean ± SD of at least three independent experiments. *** *p* < 0.005 vs. untreated basal Ctr; # *p* < 0.05, ## *p* < 0.01 vs. untreated control (C) from CM.

Interesting data emerging from the array analysis allow us to identify for the first time a novel target for these two compounds. We noticed that despite the fact that HGF release in the culture medium was not significantly altered by SR or CBD in both MDA-MB-231 and MRC5 cells (Figure 6A), its levels change after CM exposure. In particular, CM from untreated MRC5 dramatically upregulate MDA-MB-231-secreted HGF, and CM from untreated MDA-MB-231 induce a significant increase in MRC5-secreted HGF. While CM from treated MDA-MB-231 did not affect HGF secretion in MRC5, CM from SR- or CBD-treated MRC5 prevent HGF release in MDA-MB-231 culture medium (Figure 6B). HGF plays a fundamental role in tumor–stroma interactions. HGF acts in a paracrine, endocrine, or autocrine way, activating the c-Met receptor kinase in a positive feedback loop [25]. We then analyzed c-Met activation in MDA-MB-231 exposed to CM from MRC5 untreated or treated with different concentrations of SR or CBD for 24 h. As shown in Figure 6C, coherently with HGF levels, CM from untreated MRC5 induce the activation of the c-Met receptor. However, both SR and CBD prevent CM-induced receptor activation.

## 3. Discussion

For many years, both CBD and SR have been investigated for their antitumor potential. The increasing and constant attention on their potential comes from their pleiotropic effects in cancer and related mechanisms, ascribable to cannabinoid receptor (CBR)-dependent or -independent actions [11,12,13,14,15].

We previously demonstrated that in CRC, SR exerts its antitumor effect through the inhibition of p300 acetyltransferase [12,13]. This mechanism potentially explains the ability of SR to affect multiple signaling pathways in cancer and in the regulation of the tumor microenvironment. Concerning CBD, multiple mechanisms have been proposed and reviewed [11,26]. Of note, several authors suggested the potential of SR and CBD in the regulation of the tumor microenvironment, based on their pleiotropic effects [16,17,27]. However, a detailed characterization of their influence on the tumor–stroma interconnection is still required.

In recent years, the role of the TME in cancer initiation and progression has received increasing attention, emerging as a potential innovative therapeutic target. The TME plays a crucial role in supporting tumor growth, metastases formation, response to therapy, and crosstalk between stromal cells, including fibroblasts and immune cells in the metastatic niche [1,2]. Within the TME, a large number of molecules, including cytokines, chemokines, growth factors, and extracellular vesicles (EVs), contribute to the fine interaction between cancer cells and stromal components to create a favorable environment surrounding the primary tumor or, in secondary tissues, to promote pre-metastatic niche formation and metastasis [2,7,8]. In the TME, a key event promoting cancer progression is the switch of normal fibroblasts to CAFs. It is largely accepted that, through their secretome, CAFs provide structural and functional changes required for tumor progression, like ECM remodeling and metabolic and immune reprogramming [8].

The hypothesis of a direct TME reprogramming mediated by SR or CBD is supported by much data [16]. However, few studies have analyzed the specific effect of these compounds on restraining the tumor–stroma interplay and the TME composition. Elbaz and colleagues [17] reported that CBD inhibits EGF-induced breast cancer cell proliferation and, interestingly, reduces MMP9 secretion and lung metastasis in vivo. They also found that CBD prevents CM-CSF, CCL3, and MIP-2 release in culture medium from murine triple-negative breast cancer cell line 4T1.2 [17]. Of note, it has been shown that in MDA-MB-231 cells, CBD inhibits exosome and microvesicles release [28], suggesting and enforcing its role as a potential modulator of TME-associated pathogenic events, also in cancer where EVs are involved in pre-metastatic niche conditioning [29].

In this work, we demonstrated that SR and CBD directly affect several pathways involved in breast cancer progression and, in turn, modulate and rewrite the TME composition. In particular, for the first time, we characterized the effect of the two compounds on growth factor secretion from metastatic breast cancer cells and normal and activated lung fibroblasts to mimic tumor–stroma interactions during breast-to-lung metastasis processes. Interestingly, we found that non-toxic concentrations with minimal direct effect on proliferation generate beneficial bidirectional actions. We found that conditioned media from MRC5 normal lung fibroblast treated with SR or CBD prevents MDA-MB-231 cell proliferation and migration through a significant reduction in active MMP9. On the other hand, the treatment of breast cancer cells prevents lung fibroblasts from switching to the CAF-like phenotype, as proved by the reduction in α-SMA and c-Myc expression observed in MRC5 cells. In lung fibroblasts, c-Myc plays a central role in the regulation of stage-specific transcriptional plasticity during breast-to-lung metastasis [22]. Our data are further supported by the reduction in c-Myc transcriptional activation in MDA-MB-231 cells mediated by both SR and CBD.

Aiming to define a direct effect and to investigate how these compounds modify the contribution of single components in the TME, we first examined the changes in growth factor release from metastatic breast cancer cells and normal lung fibroblasts treated with our substances. Then, to analyze if the treatments, indirectly, and the modified secretome profiles, directly, impact on tumor–stroma interactions, we performed the same analyses after exposure with reciprocal conditioned media from treated cells. In this setting, the membrane array analyses provided much interesting data. In both MDA-MB-231 and MRC5 cells, SR was able to reduce growth factors widely involved in the autocrine and paracrine regulation of tumor proliferation, angiogenesis, and immune reprogramming. The finding that SR reduces EGF, SCF, and VEGF-D is in line with the decrease in HIF1α transcriptional activity. Among HIF1α responsive genes, there are several members involved in tumor growth, angiogenesis, and survival [30], and it has been demonstrated that in breast cancer cells, HIF1α directly regulates SCF gene expression also as a response to EGF [31]. Interestingly, the reduction in SCF secretion was verified also in MDA-MB-231 exposed to CM from MRC5 cells treated with SR.

IGFs, FGF, and PDGF are among the well-studied growth factors that activate pro-carcinogenic signaling pathways. It is widely accepted that PDGF/PDGFR signaling acts in an autocrine and paracrine manner to directly stimulate tumor or stromal cells. The stimulation of PDGFR by its ligands, such as PDGF-AA, PDGF-BB, PDGF-AB, and others, activates multiple pathways (e.g., MAPK, Notch, MMPs, and TGFβ) involved in tumor progression and metastasis [32]. In our models, SR and CBD reduce the secretion of different PDGFR ligands, both in cancer cells and lung fibroblasts. Moreover, also in activated fibroblasts exposed to CM from CBD-treated breast cancer cells, we noticed a reduction in PDGF-AB secretion. In SR-treated MDA-MB-231 and in MDA-MB-231 treated with CM from CBD-treated lung fibroblasts, we found a reduction in several FGF family members. The FGF/FGFR axis has received increasing interest in recent years, becoming an attractive target for therapeutic intervention also in breast cancer. This interest arises from its clear role in metastasis, resistance to anticancer therapies (including endocrine therapies), and in the interaction between tumor and stromal cells in the breast TME [33].

Membrane array analysis revealed that our compounds are able to modulate crucial players of IGF pathways, like IGFs ligands and IGFBPs. Specifically, as a direct effect in MDA-MB-231 and MRC5 fibroblasts, both SR and CBD significantly reduce IGFII secretion. On the contrary, several IGFBPs are upregulated when the cells were exposed to CM from treated cells. A previous study reports that the CBD effect on breast cancer cell proliferation can be antagonized by IGF1 [34]. The IGF signaling pathway is involved in breast cancer initiation and progression, supporting cancer stem cells, EMT, migration, and invasion [35]. Moreover, Gui and colleagues demonstrated that the protumorigenic effect of metastatic breast CAFs is associated with the increased production of IGFII [36]. Concerning the role of IGFBPs in cancer, there is still controversial evidence, particularly regarding some of them, but overall, it seems that in several cancers, including breast, they exert a tumor-suppressive role. It is generally accepted that IGFBP1, IGFBP4, and IGFBP6 inhibit tumor development and progression, and their antitumor activity is mainly ascribable to IGF sequestering [37]. Of course, future studies will be required to clarify the role of these compounds in regulating the complex dynamic between IGFs, IGFBPs, and pathway activation in breast and other cancers to understand their potential as IGF signaling inhibitors.

The results highlighted that SR and CBD are also able to modulate some factors involved in tumor immune surveillance in the TME. SR reduces the release of PIGF in both MDA-MB-231 and MRC5, while CBD inhibits its secretion only in MRC5 cells. PIGF is the ligand of VEGFR1, through which it induces cytokine production and blunts antitumor immunity. PIGF is regulated by, and in turn regulates, HIF1α and NfKB. It increases NfKB transcriptional activity, which translates into the stimulation of pro-inflammatory cytokines, Cox2, and MMP9 expression [38]. The SR- or CBD-mediated decrease in PIGF released in culture media is coherent with the reduction in HIF1α and NfKB transcriptional activation in MDA-MB-231 cells. GCSF and GMCSF belong to the hematopoietic growth factor family. In breast cancer patients, high GCSF levels have been associated with metastatic disease, and it seems that tumor-derived GCSF induces tumor growth, angiogenesis, EMT, and the stemness-like invasive phenotype [39]. Here, we found that SR and CBD almost abrogate the secretion of GCSF, and interestingly, this effect was verified also in activated fibroblasts exposed to CM from CBD-treated MDA-MB-231. Interestingly, when activated fibroblast are exposed to CM from both CBD- and SR-treated MDA-MB-231 cells, the release of GMCSF also decreases. Despite that the role of GMCSF in cancer is still debated, it is clear that in breast cancer its aberrant levels, or prolonged exposure, induces EMT and invasion and migration to distant sites [40].

The ability of our compounds to modulate the release of CSFs seems to be a very intriguing point. In breast cancer, tumor-derived GCSF promotes the generation of myeloid-derived suppressor cells able to inhibit T-cell activation and proliferation, leading to metastatic enhancement [39]. Recently, Su and colleagues demonstrated that breast-tumor-cell-derived GMCSF drives the accumulation of ARG1-expressing myeloid cells, well-known markers of inhibition of host antitumor immunity, and then dictates the acquisition of the immunosuppressive TME [41].

Moreover, interesting data from this work describe the modulation of the TGF family and the finding that SR and CBD affect HGF secretion and activity. The TGF cytokine family encompasses several members of secreted factors involved in the regulation of cancer and the TME. Acting through SMAD-dependent or -independent pathways, TGFβ signaling plays a clear role in tumor initiation and progression, invasion and metastatic niche formation, EMT, immune response inhibition, and angiogenesis [18,42]. Along with the Notch pathway, TGFβ promotes tumor growth, EMT in cancer stem-like cells, and the angiogenesis of breast cancer, in vivo [43]. In our settings, in cancer cells and lung fibroblasts, TGFβ release was inhibited by SR, CBD, and after exposure to CM. These data agree with the expression and localization of SMAD proteins, which in turn regulate promoter activity in the nucleus, as demonstrated by the luciferase assay, where both compounds reduce the SMAD-responsive element activation.

The HGF/c-Met pathway is crucial in the crosstalk between cancer and stromal cells in the TME. Stromal cells like fibroblasts and CAFs are the main producers of HGF in the TME, and the activation of the c-Met pathway occurs in a paracrine fashion, also in cancer cells [25,44]. Hyperactive HGF/c-Met signaling in cancer represents a hallmark supporting tumor growth and survival, metastasis, cancer stemness, and chemoresistance. HGF/c-Met inhibitors overcome resistance to EGFR, FGFR, BRAF, VEGF, and HER2 inhibitors [44,45]. Here, we found that, despite that our compounds did not significantly affect the release of HGF in cancer cells or in lung fibroblasts, exposure to CM strongly increases HGF levels in culture medium, in both MDA-MB-231 and MRC5, as expected. The interesting finding is that, when cancer cells are exposed to CM from treated fibroblasts, the release of HGF is strongly prevented and, coherently, the activation of c-Met kinase is reduced. This observation suggests for the first time that SR and CBD could be considered in further studies as HGF/c-Met pathway inhibitors and potentially can be used to overcome chemoresistance in breast and other cancers. On the other hand, in our previous study, we demonstrated the efficacy of SR in overcoming cancer stemness and chemoresistance in CRC [13].

Overall, the results enforced the antitumor potential of SR and CBD and provide new interesting cues on the dynamic triggered in breast cancer and the crosstalk with stromal cells in the TME. Clearly, future studies will be focused on the detailed study of their effect on selected pathways, here emerging as new pharmacological targets, to establish the connection between all the mechanisms proposed so far by several authors, which definitely underscore their promising potential.

## 4. Materials and Methods

### 4.1. Reagents and Antibodies

SR (Rimonabant, SR141716A), kindly donated by Sanofi-Aventis (Montpellier, France), and CBD (Cannabidiol), purchased from Sigma-Aldrich (St. Louis, MO, USA), were dissolved in DMSO and added to cell cultures at the indicated concentrations.

Anti-phospho-β-Catenin (Ser33/37) (#2009), anti-GAPDH (#2118), anti-phospho-NfKB p65 (Ser536) (#3033), anti-NfKB p65 (#4764), anti-phospho-SMAD2 (Ser465/467)/SMAD3 (Ser423/425) (#8828), and anti-SMAD2/3 (#3102) were purchased from Cell Signalling Technology (Beverly, MA, USA). Anti-β-Catenin (ab32572), anti-Lamin A+C (ab8984), anti-α-SMA (ab7817), and anti-MMP9 (ab119906) were purchased from Abcam (Cambridge, UK). Anti-phospho-Met (Tyr1365; sc-377548) and anti-Met (sc-514148) were purchased from Santa Cruz Biotechnology (Dallas, TX, USA). Secondary HRP-linked goat anti-mouse (ab6789) or goat anti-rabbit IgG (ab6721) antibodies were from Abcam (Cambridge, UK).

### 4.2. Cell Cultures, Cell Viability, and Cell Proliferation Assay

Human breast cancer cell line MCF7 (ERα positive) and the highly aggressive, invasive, and poorly differentiated triple-negative breast cancer (TNBC) cell line MDA-MB-231 were purchased from the ATCC (American Type Culture Collection) and cultured in Dulbecco’s modified Eagle medium (DMEM) with 4.5 g/L glucose, 10% of FBS (Fetal Bovine Serum), 2 mM L-glutamine, and 100 U/mL Pen-Strep. Normal human lung fibroblast cells MRC5 were purchased from ATCC and cultured in Eagle’s Minimal Essential Medium (EMEM) with 10% of heat-inactivated FBS, 2 mM L-glutamine, 100 U/mL Pen-Strep, and 1% Na-Pyruvate. All the cell lines were routinely grown in monolayers and maintained at 37 °C in a 5% CO_2_ humified atmosphere and regularly tested for mycoplasma presence. In all experiments, cells were treated with the substances after 24 h of incubation in the media with 2% FBS.

Cell viability and cell proliferation were evaluated through a colorimetric MTT metabolic activity assay and with an ELISA kit based on 5-Bromo-2′-deoxy-uridine (BrdU) labeling and detection (Roche Diagnostics GmbH, Mannheim, Germany), respectively, as previously described [46].

### 4.3. Western Blot Analysis

Total protein extracts were obtained by lysing cultured cells with ice-cold RIPA buffer (50 mM Tris–HCl pH 8.0, 150 mM NaCl, 1% Nonidet P-40) supplemented with protease and phosphatase inhibitors (Sigma). Subcellular fractionation was performed using NE-PER^®^ Nuclear and Cytoplasmic Extraction Reagents (Thermo Scientific, Waltham, MA, USA, Pierce biotechnology), as previously described [12]. Protein concentration was determined using the Bradford method using bovine serum albumin as standard. In total, 10–30 μg of proteins was loaded and subjected to 8–12% SDS-PAGE, under reducing conditions. Gels were electroblotted into nitrocellulose membranes that were probed with the primary antibodies. Membranes were incubated with an enhanced chemiluminescence (ECL) reagent solution (GE Healthcare, Hilden, Germany) and exposed to X-ray film (Santa Cruz, Santa Cruz, CA, USA) or to Amersham Imager 600 (GE Healthcare). Immunoreactive band density was quantified with ImageLab v4.0 analysis software (Bio-Rad, Hercules, CA, USA) or with TotalLab Quant v2.2 software (TotalLab Ltd., Tyne, UK).

### 4.4. Gene Reporter Assay

The luciferase assay was performed as previously described [12]. Briefly, MCF7 and MDA-MB-231 cells were transiently co-transfected with a firefly luciferase construct (100 ng) containing the Transcriptional Responsive Elements (TREs) of selected genes and the Renilla luciferase vector (10 ng) to normalize transfection efficiency (Cignal Finder Reporter assay kit, QIAGEN, Hilden, Germany). A non-inducible reporter construct encoding firefly luciferase under the control of a basal promoter element (TATA box), without any additional TREs, and a constitutively expressing GFP construct were used as negative and positive controls, respectively. After 24 h from transfection, cells were treated with SR and CBD (5 μM) for 18 h. Subsequently, the efficiency of transfection was evaluated in cells transfected with the positive control, and luciferase activity was measured using a dual luciferase assay system (Promega, Madison, WI, USA). Luciferase readings were measured using an EnSpire-2300 luminometer (Perkin Elmer, Waltham, MA, USA). Data were represented as relative luciferase activity, obtained by the ratio of firefly values (promoter reporter) to Renilla values (control reporter). Experiments in triplicate were repeated at least three times, and values were expressed as the mean ± SD.

### 4.5. Semiquantitative RT-PCR

Total RNA extraction, cDNA synthesis, and reverse-transcription PCR were performed as previously described [12]. Primer pairs specific to human β-Catenin (5′-GTCCGCATGGAAGAAATAGTTGA-3′ forward and 5′-AGCTGGTCAGCTCAACTGAAAG-3′ reverse) to human c-Myc (5′-ACCCTTGCCGCATCCACGAAAC-3′ forward and 5′-CGTAGTCGAGGTCATAGTTCCTGTTGG-3′ reverse) or to human actin B (5′-ACTGGGACGACATGGAGAA-3′ forward and 5′-ATCTTCATGAGGTAGTCAGTCA-3′ reverse) were used. All reactions were performed at least in triplicate and repeated at least three times; the PCR products were quantified using Quantity One 1-D analysis software (Bio-Rad), and the results were normalized to those obtained from actin B.

### 4.6. Conditioned Media (CM) Collection

Human breast cancer (MDA-MB-231 and MCF7) and normal human lung fibroblast (MRC5) cells were each cultured in their culture medium supplemented with 10% of FBS. After 24 h incubation, the media were replaced with fresh media with 2% FBS, and after a further 24 h of incubation, SR, CBD, or vehicle alone was added. After 24 h, CM were collected, clarified using centrifugation (2000 rpm for 10 min), and used for subsequent analyses (Figure 4A).

### 4.7. Transmigration Assay

The migration assay was performed using Transwell inserts with 8 μm pore filters (Corning Incorporated, New York, NY, USA). Briefly, 3 × 10^4^ MDA-MB-231 cells in serum-free DMEM were added to the upper chambers. Then, DMEM supplemented with 2% FBS or CM from MRC5 cells was added to the lower chambers as chemoattractant. For co-culture experiments, 3 × 10^4^ MRC5 cells in EMEM 2% FBS were added to the lower chambers. As a negative control, serum-free DMEM was added to the lower chambers. After incubation with SR, CBD, or vehicle alone for 24 h, the medium was removed from upper chambers, and the cells on the inside of the Transwell inserts were gently removed using PBS-moistened cotton swabs. To allow cell fixation, the Transwells were placed into 70% ethanol for 10 min. Then, cells which migrated to the lower surface of the membrane were stained with 0.1% crystal violet (Sigma-Aldrich, St. Louis, MO, USA), washed with PBS to remove unbound crystal violet, and then air-dried. The invaded and migrated cells were observed and imaged under a microscope. To quantify the cell migration, an acetic-acid-dependent elution of bound crystal violet was performed. Briefly, crystal violet was eluted using 33% acetic acid and quantified by measuring the absorbance at 590 nm with a plate reader. All experiments were performed in duplicate and repeated at least three times, and the results were expressed as the mean ± SD.

### 4.8. Analysis of Human Growth Factor Profile

A Human Growth Factor Antibody Array Membrane (Abcam, Cambridge, UK) was used according to the manufacturer’s instructions to analyze the supernatant from MDA-MB-231 and MRC5 treated with SR and CBD or with CM. Densitometric analysis of the membrane spots was performed using TotalLab Quant v2.2 software (TotalLab Ltd.), and array data normalization was performed according to manufacturer’s instructions. The experiments were performed three times, and the results were expressed as the mean ± SD.

### 4.9. Statistical Analysis

Data obtained from multiple experiments were calculated as means ± SD and analyzed for statistical significance using the two-tailed Student’s *t*-test. All data shown are representative of at least three independent experiments performed in triplicate. Values of *p* < 0.05 were considered statistically significant.

## Data Availability

Data sharing is not applicable to this article.

## Supplementary Materials

The following supporting information can be downloaded at https://www.mdpi.com/article/10.3390/ijms241713427/s1.

## Abbreviations

PDGF, Platelet-Derived Growth Factor; TGF, Transforming Growth Factor; FGF, Fibroblast Growth Factor; MMP, matrix metalloproteinase; α-SMA, Alpha Smooth Muscle Actin; FAP, fibroblast activation protein; FSP, Fibroblast Surface Protein; TCF/LEF, T-cell factor/lymphoid enhancer factor; RBP-Jk, Recombination Signal Binding Protein for Immunoglobulin Kappa J Region; SMAD, Small Mother Against Decapentaplegic; HIF, hypoxia-inducible factor; TNF, Tumor Necrosis Factor; VEGF, Vascular Endothelial Growth Factor; PlGF, Placental Growth Factor; IGF, insulin-like growth factor; IGFBP, insulin-like growth factor binding protein; GCSF, Granulocyte Colony-Stimulating Factor; MCSF, macrophage colony-stimulating factor; SCF, Stem Cell Factor; HGF, Hepatocyte Growth Factor; EGF, Epidermal Growth Factor; GMCSF, Granulocyte–Macrophage Colony-Stimulating Factor; CCL3, C-C Motif Chemokine Ligand 3; MIP-2, Macrophage Inflammatory protein-2; EMT, Epithelial–Mesenchymal Transition; BRAF, V-Raf Murine Sarcoma Viral Oncogene Homolog B; EGFR, Epidermal Growth Factor Receptor; VEGFR, Vascular Endothelial Growth Factor Receptor.
