# Supplementary material for: Rimonabant and Cannabidiol Rewrite the Interactions between Breast Cancer Cells and Tumor Microenvironment
Source: Int J Mol Sci. 2023 Aug 30;24(17):13427. doi: 10.3390/ijms241713427 (PMC10487984; doi:10.3390/ijms241713427)
Supplement: Supplementary file 1 [file ijms-24-13427-s001.zip › ijms-2564428-supplementary.pdf]

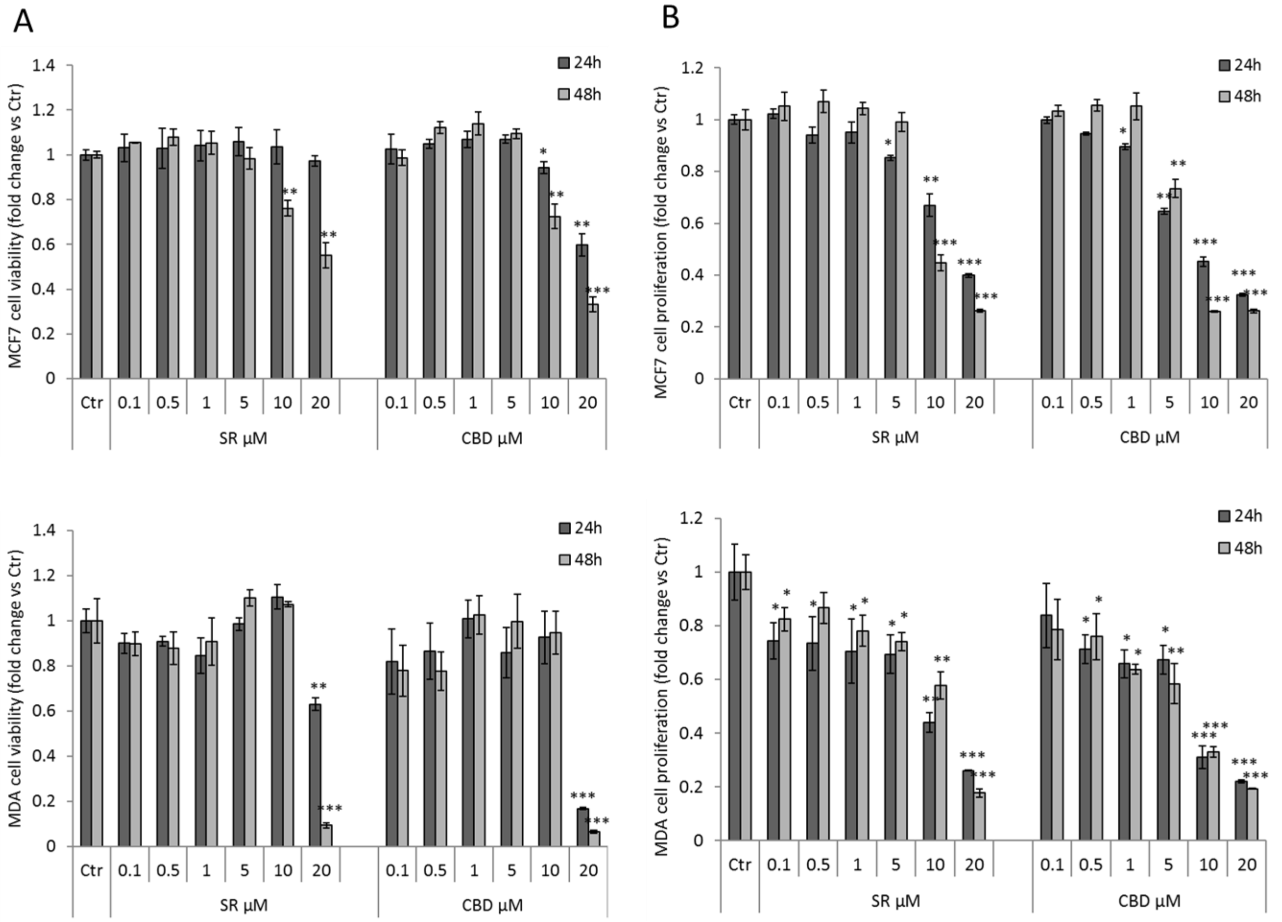

Figure S1. Effect of SR and CBD on cell viability (MTT assay) (A) and cell proliferation (BrdU incorporation assay) (B) in MCF7 and MDA-MB-231 cell lines, treated as indicated for 24h or 48h. Data are expressed as mean ± SD of at least three independent experiments. *$p < 0.05$, **$p < 0.01$, ***$p < 0.005$ vs. control.

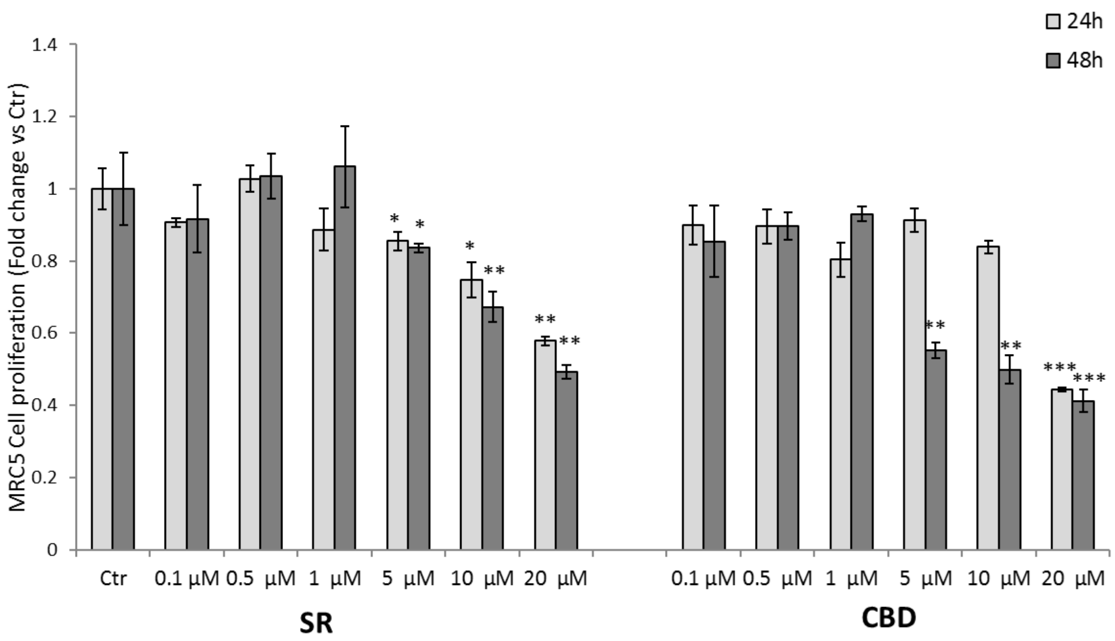

Figure S2. Cell proliferation assay performed in MRC5 cells treated with vehicle, SR or CBD for 24h or 48h. Data are expressed as mean ± SD of at least three independent experiments. *p < 0.05, **p < 0.01, ***p < 0.005 vs. control.

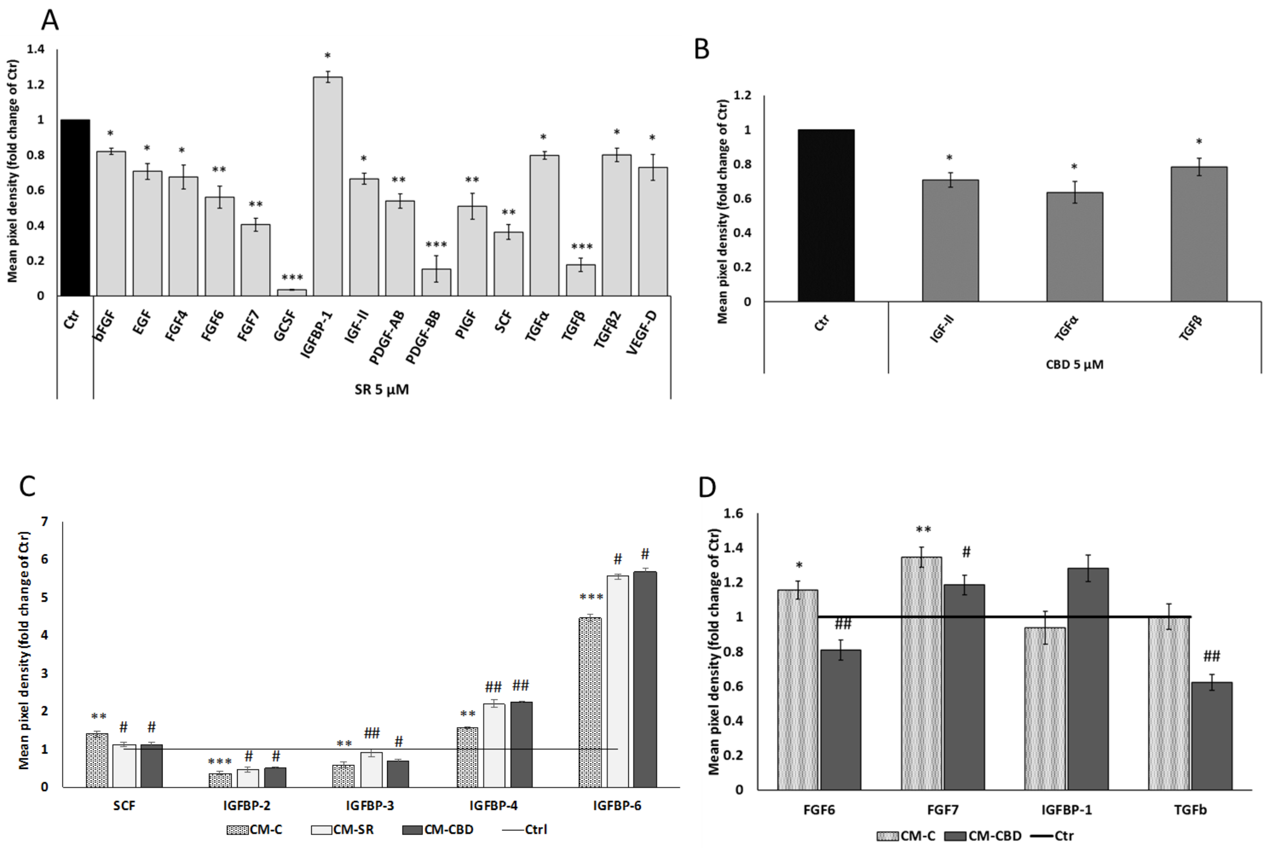

Figure S3. Quantification of human growth factors secreted in culture medium from MDA-MB-231 cells treated for 24h with SR, CBD (A, B) or CM from MRC5 (untreated or treated with SR or CBD for 24h as indicated) (C, D). Histograms represent densitometric analyses of the spots produced by antibody-target analyte complexes formed on the membrane array. Data are expressed as mean ± SD of three independent experiments. *p < 0.05, **p < 0.01, ***p < 0.005 vs. untreated basal Ctr; # p < 0.05, ## p < 0.01 vs untreated control (C) from CM.

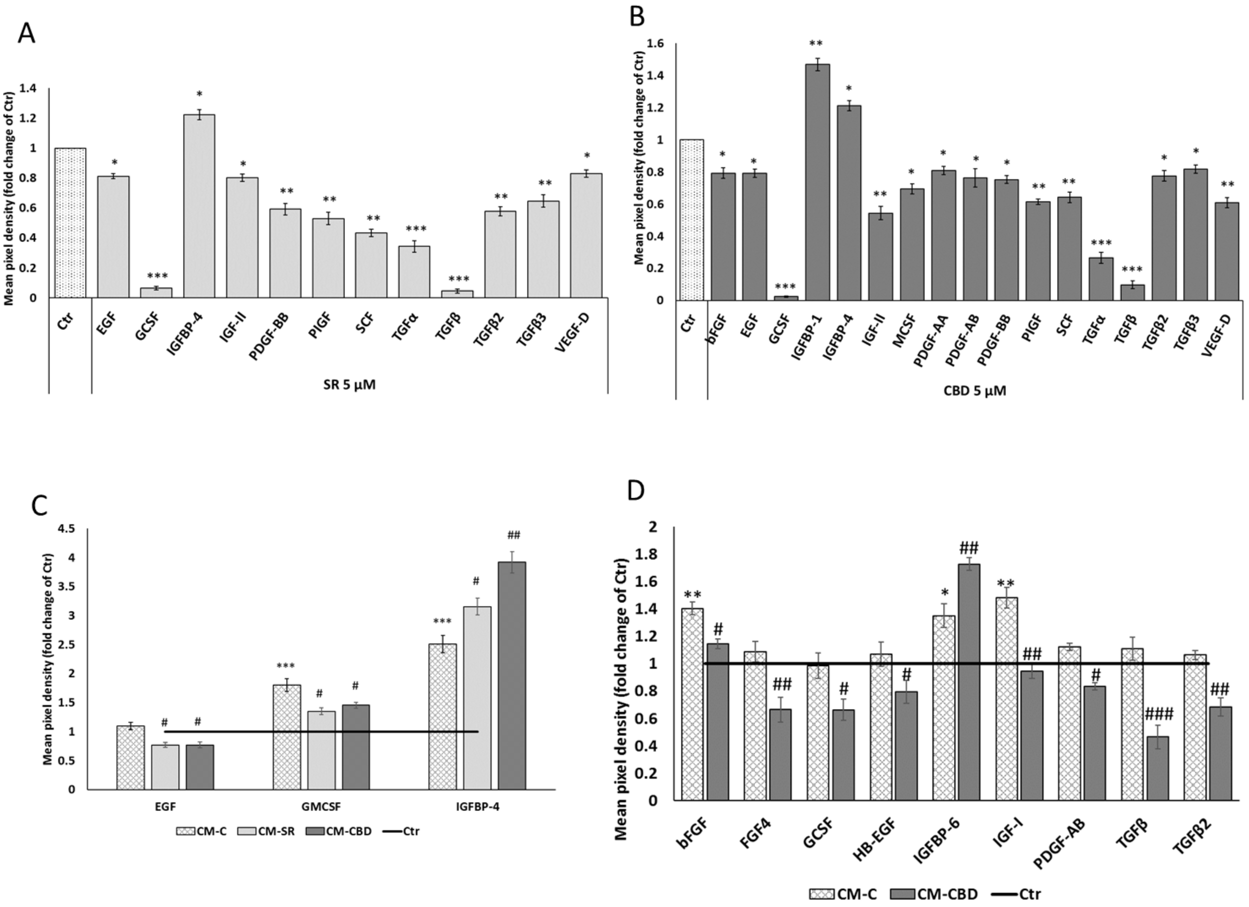

Figure S4. Quantification of human growth factors secreted in culture medium from MRC5 cells treated for 24h with SR (A), CBD (B) or CM from MDA-MB-231 untreated or treated with SR or CBD for 24h as indicated (C and D, respectively). Histograms represent densitometric analyses of the spots produced by antibody-target analyte complexes formed on the membrane array. Data are expressed as mean ± SD of three independent experiments. *$p < 0.05$, **$p < 0.01$, ***$p < 0.005$ vs. untreated basal Ctr; # $p < 0.05$, ## $p < 0.01$, ### $p < 0.005$ vs untreated control (C) from CM.
